# Team-Effectiveness Barriers and Solutions to Depression Screening within a Pediatric Hospital Network: A Qualitative Study

**DOI:** 10.1080/23794925.2026.2711444

**Published:** 2026-07-30

**Authors:** William Bevens, Lauren Brookman-Frazee, Hannah N. Edwards, Cynthia Kuelbs, Nicole A. Stadnick

**Affiliations:** a Department of Psychiatry, University of California, San Diego, CA, USA;; b Child and Adolescent Services Research Center, San Diego, CA, USA;; c UC San Diego ACTRI Dissemination and Implementation Science Center, La Jolla, CA, USA;; d Rady Children’s Hospital, San Diego, CA, USA;; e Department of Pediatrics, University of California, San Diego, CA, USA

## Abstract

**Background:**

An increasing number of hospital systems have implemented universal depression screening protocols in response to the growing prevalence of pediatric depression. However, to date no studies have qualitatively explored how the sustainment of depression screening workflow functions in practice.

**Objective:**

To characterize the workflow for an existing universal depression screening pathway within a large pediatric healthcare system and explore barriers and solutions to barriers in implementing the workflow effectively.

**Method:**

Eight focus groups with 37 staff members in a large pediatric hospital system were conducted. Participants were considered eligible if they were involved in depression screening within their unit. Focus groups were recorded, transcribed, and analyzed for themes using rapid qualitative analysis.

**Results:**

Six barriers and five proposed solutions were identified as common themes across units, organized along three stages of the depression care cascade: screening, assessment and resources, and disposition planning. Key barriers included the language appropriateness of the Patient Health Questionnaire-9 (PHQ-9) for younger and neurodivergent patients, siloing of medical and behavioral health roles, and lack of infrastructure for follow-up after referral. Proposed solutions included adapting screening language, cross-training staff in mental health assessment, and designating dedicated roles for follow-up.

**Conclusions:**

Our findings suggest that strategies aimed at improving how teams work together could assist hospitals in delivering depression screening at all stages through better team coordination and role clarification.

## Introduction

The lifetime prevalence of depression in children aged 3–17 in the United States has increased by between 3.1% and 5.4% annually from 2016 to 2022 and now represents an estimated 5.3% to 7.1% of all children (Casseus & Reichman, 2025; Leeb et al., 2024). The growing prevalence of childhood depression has been attributed to several factors, such as familial economic stress (Mojtabai et al., 2016; Weinberger et al., 2018), social media use (Masri-Zada et al., 2025; Saleem et al., 2024; Twenge et al., 2018), and changes to mental health screening (Keyes et al., 2019; Mojtabai et al., 2016), the last of which has become the focus for health system policymakers in recent times. In this context, the United States (US) Preventive Services Task Force first recommended universal screening for major depressive disorder in adolescents aged 12 to 18 years in 2016 and again in 2022 (Siu & US Preventive Services Task Force, 2016; US Preventive Services Task Force, 2022).

Since the USPSTF recommendation, various iterations of pediatric depression and suicide screening protocols have been implemented across whole pediatric hospital systems (Crandal et al., 2022) and in pediatric clinical settings within the US, such as primary care (Bose et al., 2021; Chowdhury & Champion, 2020; Davis et al., 2021; Harder et al., 2019; Kilkelly et al., 2023; Siniscalchi et al., 2020), specialty clinics (Iturralde et al., 2017; Liu et al., 2020; Marker et al., 2019; Matthews et al., 2025; Quittner et al., 2020), and urgent care (Patel et al., 2018). While the implementation of depression screening has demonstrated mostly positive outcomes related to treatment linkage across these settings (Bose et al., 2021; Chowdhury & Champion, 2020; Kilkelly et al., 2023; Siniscalchi et al., 2020), several challenges associated with sustainment were identified, including workflow constraints, limited capacity for follow-ups and referrals, and lack of local champions (Liu et al., 2020; Quittner et al., 2020).

Specific to the hospital system level, there are limited data on the implementation of universal depression screening despite high volumes of child and adolescent patients accessing this system annually (Cairns et al., 2023). The only large-scale example is a comprehensive pediatric healthcare system in southern California that implemented universal depression screening for all patients aged 12–17 years starting in 2016 (Crandal et al., 2022). This system screens adolescents across inand outpatient specialty/medical care units, the emergency department (ED), and urgent and primary care settings. Despite this, implementation gaps were reported, including differential rates of screening across clinics and significant uncertainty around service linkage and treatment access for patients due to referrals from positive screens only being tracked to internal services. Importantly, this study reported that 87.6% of patients who screened positive for depression presented to the ED did so for a non-psychiatric concern, which highlights the importance of the hospital system to the pediatric behavioral health system in the US. As such, an exploration of current practices is needed to elucidate gaps within the depression care cascade across units within this hospital system.

The depression care cascade represents the steps within the clinical care framework for depression screening (Pence et al., 2012), such as screening, diagnosis, treatment initiation and response to treatment. Conceptualizing depression screening and linkage as a care cascade allows researchers to sequentially describe barriers to delivering universal depression screening, explore solutions to these barriers, and identify implementation strategies needed to support successful implementation and sustainment. Building on this framework, the present study explores universal depression screening within a large pediatric healthcare system that has implemented screening across multiple specialty departments. By characterizing existing workflows and the challenges staff encounter during routine depression screening, we aim to provide data for hospital systems seeking to implement or refine similar implementations. Specifically, this study aims to:

1. Examine and describe current universal depression screening workflows across five specialty departments already implementing universal depression screening in a large pediatric healthcare system.
2. Identify implementation challenges and potential solutions to inform efforts to improve implementation of universal depression screening across hospital systems.

## Methods

This study took place between July 2024 and June 2025 and is embedded within the National Institute of Mental Health Advanced Laboratories for Accelerating the Reach and Impact of Treatments for Youth and Adults with Mental Illness Center [Masked] that supports research on how team-based implementation strategies could improve services for children with mental health and developmental needs. All research activities were approved by the UC San Diego Institutional Review Board. The study protocol has been published previously [Masked]; a brief overview of this study’s methods is described below.

### Study design

#### Participants and setting

A total of eight focus groups were conducted with three medical outpatient units, the Emergency Department, and one medical inpatient unit. These focus groups occurred in two phases and averaged 48 minutes in duration. Phase 1 included five focus groups (median [IQR] participants per group = 6 [6–7]; *n* = 29) with physicians and allied health professionals from four units. Phase 2 included three focus groups (median [IQR] participants per group = 2 [2–4]; *n* = 8) with mental health providers who are assigned to four medical units.

Units with high rates of depression screening and/or mental health referrals based on administrative data from the past year were selected for inclusion in this study. This decision was made to maximize learning of current screening practices, whereby units with the highest volumes of screenings would likely have the greatest number of lessons learned and feedback to provide. Invitations to the focus groups for this study were coordinated by leaders within each unit who were given autonomy to determine who they would invite with consideration of our study aims (i.e., to examine and describe current universal depression screening workflows and identify implementation challenges and potential solutions). In this context, unit leaders were best equipped to determine who were the most relevant personnel to support our data collection activities. Unit leaders then notified the research team, which sent formal recruitment e-mails to all eligible staff. Two units recommended that invitations be sent to all their personnel while two nominated fractions of their personnel.

Upon review of findings from the first focus groups with physicians and staff, we determined that additional insights from mental health providers responsible for later steps in the depression screening cascade (i.e., referrals following a positive screen) were needed for the workflow analysis undertaken. The research team then reached out to the manager of the hospital-wide Social Work department assigned to the units interviewed in Phase 1 to organize Phase 2 focus groups. Data from 37 participants across five units were included in this study (Table 1).

### Procedure

Interview guides (Supplementary Table S1) were developed by the study team using an adapted brainwriting premortem technique (Gilmartin et al., 2019). Brainwriting premortem combines asynchronous brainstorming in a group setting (Paulus et al., 2015) with a “premortem” exercise that references the fact that the group discussion is conducted *before* implementation of a program (“pre”). Participants are presented with a scenario that assumes that an identified program failed (“mortem”) (Klein, 2007) so participants are requested to reverse engineer why the program failed and then preemptively suggest possible solutions. We adapted this method by anchoring the “premortem” in the established universal depression screening protocol operationalized by each unit and then inviting participants to identify challenges in its historical and current implementation. These focused on four topics: 1) current protocol for depression screening, linkage to care, and tracking outcomes; 2) elements of a successful depression care cascade team; 3) challenges in team communication related to the depression care cascade workflow; 4) possible solutions to address each challenge. Focus groups took place virtually via Zoom, and both audio and video were recorded verbatim using Zoom’s native recording features. Informed consent was obtained in advance and reviewed at the beginning of each focus group session. NS and WB led all interviews using a semi-structured approach, which allowed facilitators to probe interviewees to clarify, add depth, or contextualize their responses. HE and LBF observed and took detailed, real-time field notes within the summary template (Supplementary Table S2). This summary template contained questions on the column headers and participant roles and names on the row headers. The research team reviewed all summary templates after each focus group and subsequently sent them to participants for member-checking and to ensure accuracy. Focus groups were transcribed using Rev.com. All transcripts were checked for accuracy by HE.

#### Data analysis

Clinical workflow analysis was used to explore the current depression screening protocol (Palinkas & Zatzick, 2019; Sheehan & Bakken, 2012; Wu et al., 2022). This analysis characterized usual care processes in each unit by mapping workflow activities to specific job titles within a unit. Data were then visualized to assist with specifying communication pathways between roles, which were used to identify “pain points” related to screening and service linkage. These visualizations were presented to participants in the Phase 2 focus groups to orient social workers to the pathways described within focus groups in Phase 1.

Rapid qualitative analysis (RQA) was used to analyze, organize, and present findings of the focus group data (Hamilton, 2013). RQA is a constructivist approach, and it was used by WB and HE to inductively generate themes within each individual unit based on the summary template generated previously (Taylor et al., 2018). WB, NS, LBF, and HE then met to discuss and refine the themes generated; this process was reflexive, and agreement was based on consensus. NS and LBF are clinical psychologists experienced in health services research in child and adolescent populations, WB has experience qualitative and quantitatively exploring mental health in disabled populations, and HE is experienced in supporting health services research. No researchers had a relationship with any participants beyond the process of recruitment for this project, and these interactions had no bearing on the research process undertaken. This reflexive process enabled researchers to contextualize these data in their own experiences and interrogate their biases during theme generation. Following this, common themes were consolidated to report “shared barriers” experienced across all units, which required these themes to be present in all summary templates after consensus. This analytic approach is in accordance with RQA’s template analysis origins, which prescribes that determining the salience of a theme is associated with the frequency of its underlying data across units of measure (i.e., clinics) (Hamilton, 2013). This differs from reflexive thematic analysis where the salience of a theme does not require a “threshold” of code prevalence within the dataset.

These barriers were then inductively categorized into three distinct stages of the depression care cascade: (1) screening; (2) assessment and resources; (3) disposition planning. Proposed solutions to barriers in the initial template were analyzed and organized according to the themes with which they most closely associated. These final shared barriers, solutions, and categories were then discussed and agreed upon by WB, NS, HE, and LBF. Results are presented in the format of barrier and then the associated solution if one was presented.

## Results

**Aim 1**: *Examine and describe current depression care cascade workflows across five specialty departments already implementing a depression cascade in a large pediatric healthcare system*.

Out of 96 total invitations sent out across all five units, 37 total participants participated in focus groups and provided details on the depression care cascade workflow in their unit (37/96 = 38.5% recruitment rate). Clinicians (physicians & nurse practitioners) were the largest group represented within these data, followed by social workers. See Table 2 for the care cascade steps and assigned leads reported by participants for each step across the phases of the care cascade (1) screening; (2) assessment and resources; (3) disposition planning.

Overall, roles were consistent across units for Steps 1, 2, and 6. Roles differed on Steps 3, 4, and 5 depending on the availability of a social worker to help support further assessment, conduct safety planning when indicated, and place referrals to appropriate services.

**Aim 2**: *Identify implementation challenges and potential solutions to inform efforts to improve implementation of the depression care cascade*.

Six barriers and five solutions were organized within six themes organized by the three stages of the depression care cascade. Themes are presented by stage alongside illustrative quotes (Table 3).

### Screening

#### Theme 1: language appropriateness of the PHQ-9 for younger patients

Not all adolescents, especially 12 years olds, will have the cognitive abilities to understand what the PHQ is asking.

(Medical Assistant 1)

##### Challenge:

Participants perceived that the wording of the PHQ-9 can be confusing to patients who may misunderstand the language or intent. This was mostly highlighted for younger patients (i.e., those closer to age 12), and participants reported that the age that patients presented affected how they chose to administer the questionnaire. Participants reported that younger patients particularly struggled with the words “down” and “depressed” in the PHQ. This meant that participants attempted to offer alternative explanations for these words (e.g., substituting for words like “sad” or asking if they “want to come home and cry”).

##### Potential Solutions:

Participants suggested rewording challenging questions with age-appropriate and accessible language within the questionnaire to assist patients completing it independently. It was also suggested to give the staff or provider administering the questionnaire permission and guidance on how to adapt language to ensure patient comprehension.

#### Theme 2: administering the PHQ-9 to neurodivergent patients

I put either decline or not able to do due to autism or needs assistance unable to do in private.

(Medical Assistant 2)

##### Challenge:

Participants expressed that there is missing guidance on a standardized practice for administering the PHQ-9 to patients with varying cognitive and developmental abilities. Some reported that for certain recorded diagnoses (e.g., autism), the PHQ-9 may not be administered. Others reported that they tried to administer the PHQ-9 to all patients to give them an opportunity to express their concerns and needs.

##### Potential Solutions:

No potential solutions shared.

#### Theme 3: setting for completing the PHQ-9

Medical assistants say that parents will ‘hover,’ or answer questions if the child is young.

(Nurse Practitioner 1)

##### Challenge:

The current depression screening protocol encourages patients to complete this questionnaire by themselves to facilitate honest answers; however, participants explained that parents may engage in various ways while their child completes the PHQ-9 that can be challenging. Participants observed that the presence of a parent next to a patient may influence how a patient answers a question. Additionally, staff reported that they are often required to help patients understand certain questions, which may impact the validity of their responses.

##### Potential Solutions:

Participants reported that reinforcing the importance of a child independently completing the PHQ-9 to parents and asking them to leave the room may help. Depending on the unit and its environment, staff doing intake can try to create a “safe space” for families and the patient, which may in turn make it easier for parents or caregivers to leave the patient to complete it independently.

### Assessment and resources

#### Theme 4: siloing of medical & mental health roles

Some team members do better with [mental health] patients than others in that some may better initially navigate a patient, some will grab a doctor’s attention for patient.

(Nurse 1)

##### Challenge:

Participants reported that attention to mental health can be variable depending on the individual staff members involved and their level of comfort or familiarity with managing mental health challenges. While physicians reported that they can support patient assessment and safety planning, they often consult with social workers, even in patients presenting with lower depression acuity. Participants shared that often medical issues were first prioritized, which could sometimes lead to delays in consultations for high PHQ-9 scores that delayed patient discharge (specific to the inpatient/emergency care settings).

##### Potential Solutions:

One solution proposed was to give staff additional psychiatric assessment training to promote integration of medical and mental health needs. Another proposed solution was creating a new team or position that dealt directly with further mental health assessment after a positive screen.

#### Theme 5: challenges with mental health referral & coordination

Physicians do have a pathway to refer to [the] developmental-behavioral health [unit], but even with Masked, wait list for [mental health services] is over a year long.

(Social Worker 1)

##### Challenge:

Participants reported that referrals to mental health were challenged by long waitlists preventing patients from being able to access these services. Staff maintained a running list of community-based resources; however, availability varied and was not always up to date.

##### Potential Solutions:

Participants suggested that further developing partnerships with external service providers could increase access. It was noted that this had been previously instituted in this system and overseen by a participant but requires ongoing maintenance to keep updated.

### Disposition planning

#### Theme 6: follow-up to determine service linkage

I don’t know how we fix it, but I do know that we need to have some sort of follow-up to close the loop, to make sure that the family and patient is still doing okay.

(Nurse 2)

##### Challenge:

Although there is variability by unit in the requirement to ensure service linkage, following up with patients to check for mental health service linkage after referral was reported as difficult due to lack of systems and infrastructure to track patients. This was attributed, in part, to no clinic staff having a designated role to follow up with patients about service connection.

##### Potential Solutions:

Participants indicated that having access to a dedicated staff member or an entire team that was tasked with following up with patients would make this possible. It was suggested that this could either be a new role, or the task could be added to an existing member’s position description. A prior program using mental health coordinators was able to successfully follow up with patients from an outpatient clinic. However, this program had since been dissolved.

## Discussion

This study qualitatively explored barriers and possible solutions to conducting universal depression screening from the perspectives of on-the-ground clinical staff and providers across a subset of several units within the context of a system wide implementation in a large pediatric health network in Southern California. Focus groups demonstrated that barriers existed across three stages of the depression care cascade: screening, assessment and resources, and disposition planning. PHQ-9 administration and assessment were perceived to vary depending on the staff in charge of administration and the clinical presentation of the patient. There was also a perception that medical and mental health care were separate, and the former typically took precedence in some contexts. Finally, care continuity was a challenge where there was a lack of infrastructure and staffing to follow up with patients and ensure care linkage. In the subsequent sections, we weave in clinical implications for research and practice based on our findings.

Our findings suggest that a universal pediatric depression screening protocol requires consideration of potential adaptations needed to respond to staffing workflows, characteristics and patient presentations. For example, participants in our study reported that they occasionally reworded or rephrased items for younger patients who had issues interpreting questions, whether the screener was being administered verbally or completed independently on a tablet. Evidence-based practices may be modified to meet the developmental level of a patient where complex concepts can be understood using different language or through supplementary activities (Aarons et al., 2019; Barnett et al., 2019; Weitzman et al., 2025) The cycle of monitoring and feedback is an important component of implementation sustainability (Benzer et al., 2015), which can ensure continued fidelity of screening, particularly when coupled with training to address or reinforce areas that may need extra attention to support adherence (Curry et al., 2013).

We also report that patients were perceived to struggle to understand the wording and intent within the PHQ, specifically Question 2 (i.e., Feeling down, depressed, or hopeless). Similar challenges have been reported in pediatric populations using the modified PHQ-9 for adolescents, the Patient Health Questionnaire Adolescent (Mkhize et al., 2025; Thompson et al., 2025). However, these studies occurred in lower-resourced countries, in non-hospital settings and using the alternate PHQ measure, which limits generalizability to our study. In a hospital setting but with a large adult population, the terms “down,” “depressed,” and “hopeless” were parsed as three separate symptoms (Malpass et al., 2016), which may be a similar mechanism to explain what was observed in our study. As it stands, there is a gap in the literature in our understanding of the specific nature of these comprehension difficulties associated with the delivery of the PHQ-9 for pediatric populations within healthcare settings in higher-resourced countries. While these data indicate an age-specific barrier to administering the PHQ, sociocultural factors may also play a role in comprehension difficulties that must be considered during implementation (Harry et al., 2021). Despite this, multiple studies have validated the use of PHQ for the adolescent population ages 12 and up (Allgaier et al., 2012; Fonseca-Pedrero et al., 2023; Ford et al., 2020; LaLonde et al., 2025; Richardson et al., 2010), and our data suggest that younger adolescents in the validated age range may need more support to meaningfully respond to some items. Regardless, use of the PHQ-A instead of the PHQ-9 may assist younger patients with understanding the questionnaire.

One important consideration is that despite originally being designed as a paper-and-pencil measure, the PHQ is commonly administered verbally in our study setting, which may affect its psychometric properties (Batra et al., 2025). This is informed by prior work, which suggests that even minor changes to wording, phrasing, or tone can have significant impacts on outcomes (Ford et al., 2020). In a practical context, it may be that patients in our study setting, who have literacy challenges or neurodiversity needs, are more likely to prompt staff to verbally administer the measure. Our data indicate an opportunity to leverage team effectiveness research to improve PHQ screening implementation by ensuring screening is standardized with flexibility across clinics, which could increase fidelity and service linkage.

There was significant variability in how staff determined the appropriateness of administering the PHQ to adolescents with autism. Depression has been documented as a common co-occurrence in autistic adolescents (De la-Iglesia & Olivar, 2015; Pezzimenti et al., 2019; Rai et al., 2018; Rosenberg et al., 2011), and as of 2020 the American Academy of Pediatrics recommends regular depression screening for adolescents with autism starting at age 12 (Hyman et al., 2020). Despite this, evidence suggests that autistic adolescents were still less likely to be screened for depression at child-wellness visits, yet when screening was completed, they were more likely to endorse depression and suicide risk (Hamdan & Bennett, 2024). In our focus groups, some participants shared variability in administering the PHQ to autistic patients and/or based on the individual adolescent’s presentation and cognitive ability. Other work at a university-affiliated developmental center revealed a similar gap in depression screening for adolescents with developmental differences where only 58% of individuals were screened for depression and of those, 10% had a moderate to severe score (Valicenti McDermott et al., 2024). These data suggest that despite intention, depression screening is not universally administered to neuro-diverse youth compared to rates in the total population across various settings (Crandal et al., 2022; Davis et al., 2022; Harder et al., 2019; Marker et al., 2019).

Social workers reported that medical staff increasingly relied on them for mental health assessments, including for patients with mild presentations. This reliance was perceived by social workers as impacting the capacity of the limited pool of mental health providers to address higher acuity patients. In this context, siloing represents a separation of medical and mental health tasks across roles that are intended to be integrated within existing workflows. In our study, nurses and physicians described that medical tasks were prioritized during encounters, often as a necessity. This created an increased workload for mental health providers, and often delayed downstream mental health tasks (e.g., further assessments and referrals to resources) that required mental health staff.

The identified siloing of medical and mental health teams represents a key challenge associated with Multiteam Systems (MTS), which is an organizational form where multiple teams work interdependently toward the accomplishment of collective goals (Mathieu et al., 2002). Traditionally, MTSs in the medical context involve a unique hierarchical arrangement where there is a sequential distribution of responsibilities as a patient moves from one clinical unit to another (DiazGranados et al., 2014). The context within our study deviates from this arrangement because separate medical and mental health teams communicate within the same clinical unit yet operate sequentially, relatively autonomously, and have different leadership structures. This created a situation previously described in other settings where cross-boundary MTSs may have disproportionate compositions (i.e., some teams are over or under-represented) (Davison & Hollenbeck, 2012), and the goals of the larger team [the medical team] take precedence over the smaller team [the mental health team] (DiazGranados et al., 2014). The inclusion of boundary-spanning roles and/or cross-team structures may overcome these barriers to siloing (Shuffler & Carter, 2018), particularly in a sequential system such as ours where one team’s action or inaction can influence downstream processes.

Another common challenge was the lack of follow-up with patients who had been referred to mental health services, particularly outpatient services. Other studies have described that the documentation of referrals and their outcomes is often limited or lacking, which can lead to patients not receiving timely or appropriate mental health care (Chowdhury & Champion, 2020; Marker et al., 2019). In our study, difficulty with follow-up was attributed to a lack of IT infrastructure to support staff in accessing patient referral data. In our study, staff also remarked that there is no specific team member in charge of follow-ups, resulting in inconsistency in the person following up and follow-up completion. This is parallel with the prior challenge of “siloing” where certain tasks were not assigned and defined to a specific role, which often meant that they were not completed. Importantly, follow-up to ensure service linkage is variably required as part of the universal depression screening protocol within the Masked hospital system, which may contribute to the difficulty in its implementation. Poor follow-up post-positive depression screening is the primary line of criticism toward implementations of pediatric depression screening (Beck et al., 2024), where screening for depression has limited utility when pathways to treatment remain unavailable.

Poor follow-up may also be explained by a reluctance from providers to initiate and by parents to accept referrals for mild-to-moderate cases (e.g., low depression score, low suicide risk) (Davis et al., 2021). Physicians in our study reported that they often further assessed patients with low PHQ-9 scores (or deferred assessment to mental health providers) to ensure that patients could access referrals if desired. Despite this, physicians voiced primary concerns around the availability of internal and community mental health services, which has been a growing concern for children and adolescents in the Masked region (Cama et al., 2017). In addition, participants in our study voiced that some parents have expressed apprehension toward depression screening, particularly if their child was already connected to mental health care. It is possible that parents felt that focusing on their child’s mental health would take away attention from a medical issue that was the reason for their appointment/admission. In prior work, parents have reported being apprehensive to pursue follow-up due to inconvenience or not believing it was necessary (Davis et al., 2021).

Our study has notable strengths and limitations. A primary strength is the inclusion of multiple clinical departments and a breadth of provider and staff roles central to the depression care cascade. This affords a wide and detailed lens through which to understand pain points and improvement opportunities. The first limitation is that we interviewed only a subset of clinical staff and providers within the large healthcare system implementing the depression screening protocol. There may be additional bias introduced by the nature of participant selection, which was not random, and instead purposive sampling was used by unit leadership to identify the most appropriate participants based on their involvement with depression screening. These data are not reflective of healthcare leadership involved in overseeing the overall protocol implementation. Another limitation is that we did not include patient or parent perspectives in this study. Including patient perspectives could have contextualized our results in terms of the impact of these barriers. Also, considering that parents are an important part of the depression care cascade, future studies should investigate their experiences across all stages to ensure these challenges can be overcome. Finally, our sample is comprised of clinics that first implemented universal depression screening almost a decade ago and those with high rates of positive depression screens. While this increased our ability to gather data on the barriers and facilitators of depression screening due to these clinics’ having greater experience with the protocol, our data may not generalize to clinics that newly implemented universal depression screening or those with lower rates of positive depression screens.

### Clinical implications

This study provides findings that are immediately actionable for hospital-based clinical practice where routine pediatric depression screening occurs. Specifically, the need for team-based approaches to improve team member coordination within clinics can ensure that care is consistent across providers regardless of patient presentation; improved coordination can further prevent the siloing of mental health care across care teams within clinics. Additionally, extra training and resources dedicated to servicing patients with extra needs (younger, children with autism) would ensure that they are receiving the most appropriate mental health support while in hospital. Finally, service linkage post-screening remains a critical gap for mental health care within this setting, and hospitals should dedicate resources to supporting families in accessing community resources upon discharge. This study took place within a high-resourced hospital network, and therefore, adaptations to described solutions may be required to meet the needs of smaller or resource-limited settings. These may include community partnerships rather than dedicated follow-up roles; integrated care settings may focus on formalizing communication pathways rather than cross-training; and settings serving linguistically or culturally distinct populations will require validated translations and culturally responsive follow-up protocols.

## Conclusion

This study identified several barriers to and facilitators of universal depression screening across several medical units with a large pediatric hospital system. Difficulties associated with PHQ-9 administration to younger and developmental different patients, the siloing of medical and mental health care across roles and limited systems for follow-up and care linkage were noted barriers. Future research and practice may consider team-effectiveness research to understand its role in improving coordination and role clarity across teams to ensure more integrated care among medical and mental health teams and members.

## Supplementary Material

Supp 1

Supp 2

Supplemental data for this article can be accessed online at https://doi.org/10.1080/23794925.2026.2711444

## Figures and Tables

**Table 1.** Participant number and proportion by role.

Role	Count (%)
Administrative Staff	3 (8.2)
Nurses/Nurse Practitioners	5 (13.5)
Medical Assistants	7 (18.9)
Social Workers	9 (24.3)
Physicians	13 (35.1)
Total	37 (100.0)

**Table 2.** Depression care cascade workflows.

Screening Cascade Phase	Screening Cascade Step	Unit 1	Unit 2	Unit 3	Unit 4
Screening	(1) Administer PHQ	Medical Assistant	Medical Assistant	Medical Assistant	Medical Assistant
	(2) Notify Team About Positive PHQ Screen	Medical Assistant	Medical Assistant	Medical Assistant	Nurse
Assessment & Resources	(3) Assess after Positive PHQ Screen	Physician or Social Worker	Physician	Physician	Physician
	(4) Discuss Treatment Plan, Including Safety Plan (if applicable)	Physician	Social Worker	Social Worker	Physician or Social Worker
	(5) Place Referrals (if applicable)	Physician	Social Worker	Physician or Social Worker	Physician or Social Worker
Disposition Planning	(6) Discharge	Physician	Physician	Physician	Physician

**Table 3.** List of themes and illustrative quotes from focus groups.

Theme	Quotation
(1) Language Appropriateness of the PHQ-9 for Younger Patients.	“Not all adolescents, especially 12 years olds, will have the cognitive abilities to understand what the PHQ is asking.” (Medical Assistant).
(2) Administering the PHQ-9 to Neurodivergent Patients.	“I put either decline or not able to do due to autism or needs assistance unable to do in private.” (Medical Assistant).
(3) Setting for Completing the PHQ-9.	“Medical assistants say that parents will ‘hover,’ or answer questions if the child is young.” (Nurse Practitioner).
(4) Siloing of Medical and Mental Health Roles.	Some team members do better with [mental health] patients than others in that some may better initially navigate a patient, some will grab a doctor’s attention for patient.” (Nurse).
(5) Challenges with Mental Health Referral and Coordination.	“Physicians do have a pathway to refer to [the] developmental-behavioral health [unit], but even with Masked, wait list for [mental health services] is over a year long.” (Social Worker).
(6) Follow-Up to Determine Service Linkage.	“I don’t know how we fix it, but I do know that we need to have some sort of follow-up to close the loop, to make sure that the family and patient is still doing okay.” (Nurse).

## Data Availability

Data is available upon request from the primary author.
