# Supplementary material for: Team-Effectiveness Barriers and Solutions to Depression Screening within a Pediatric Hospital Network: A Qualitative Study
Source: Evid Based Pract Child Adolesc Ment Health. Author manuscript; Available in PMC 2026 Aug 11. (PMC13456526; doi:10.1080/23794925.2026.2711444)
Supplement: Supplementary file 1 [file NIHMS2200715-supplement-Supp_1.pdf]

Aim 1 Providers and Staff Interview Guide

Developing and Testing a Team Communication Training Implementation Strategy for Depression Screening in a Pediatric Health Care System

Introduction

Thank you for joining us today. My name is [INTERVIEWER NAME] and I am a research team member of the Depression Care Cascade Study. Today you will be asked to share about the current depression care cascade workflow in your setting, map workflow activities to specific roles of team members, and identify specific "pain points" related to screening frequency and time to service linkage. Before we begin, I'd like to review the information sheet and audio consent that was sent to you. (Bring up 2 documents on shared screen).

Did you have a chance to review these documents?

- *If Yes – Do you have any questions about the information sheet?*
- *If No – The Information Sheet on the screen reviews what you will be doing today. Please read through it and share any questions.*
- *Now, please read through the exempt information sheet and let me know if you agree or disagree to items 1-6.*

This interview will be recorded. You can decline to answer any question and request that the recording be stopped at any time. The recording will only be accessible to our research team. Your responses will be summarized and combined with responses from other interviews.

Now let's go over what you will be doing today. This interview will take 60 minutes and you will receive a meal for your time. Do you have questions before we begin and start recording?

We will start the recording now.

Let's start with introductions. Please share your name, role, and experience with depression screening and linkage to care.

Now, we'll move on to questions about the current depression care cascade in your setting. By care cascade, please think about the process from screening, following up on a positive screen, referral, and linkage to care. For these questions, please also think about your care setting and what you have oversight over.

1. Please describe your current protocol for depression screening, linkage to care, and tracking outcomes.

Team Effectiveness in the Depression Care Cascade

2. How would you describe a successful depression care cascade team?

a. What does communication look like in a successful depression care cascade team? *Probe for communication in Electronic Health Record and via email or phone.*

b. What are the factors that make it more likely for team members to be on the same page versus not?

c. How do you know a depression care cascade workflow is successful?

d. Which other departments or clinical teams do you communicate depression care cascade (including safety assessments) information with?

Challenges and Opportunities for Improvement

Take 5 minutes to think about the major challenges you face in team communication about the depression care cascade workflow in your setting. Please have your top challenge ready to share.

3. Let's walk through your top challenge or pain point. For each, I'll ask you to propose one or more possible solutions that have worked or that you are newly suggesting.

- Let's start with the important challenge or pain point from your perspective?
  - Do you have suggestions or ideas about how to avoid or address this?
- Let's move on to another challenge? What is that?
  - Do you have suggestions or ideas about how to avoid or address this?

Continue this line of questioning until everyone has shared.

<u>Closing</u>*: "Thank you very much for sharing your valuable insight with me today. I will now stop recording."*

Aim 1 Mental Health Providers Interview Guide

Developing and Testing a Team Communication Training Implementation Strategy for Depression Screening in a Pediatric Health Care System

Introduction

Thank you for joining us today. My name is [INTERVIEWER NAME] and I am a research team member of the Depression Care Cascade Study. Today you will be asked to share about the current depression care cascade workflow in your setting, review a map of workflow activities to specific roles of team members, and identify specific "pain points" related to screening frequency and time to service linkage. Before we begin, I'd like to review the information sheet that was sent to you. (Bring up the document on shared screen).

Did you have a chance to review this document?

- *If Yes – Do you have any questions about the information sheet?*
- *If No – The Information Sheet on the screen reviews what you will be doing today. Please read through it and share any questions.*

This interview will be recorded. You can decline to answer any question and request that the recording be stopped at any time. The recording will only be accessible to our research team. Your responses will be summarized and combined with responses from other interviews.

Now let's go over what you will be doing today. This interview will take 60 minutes, and you will receive a meal for your time. Do you have questions before we begin and start recording?

We will start the recording now.

Let's start with introductions. Please share your name, role, and experience with depression screening and linkage to care.

Now, we'll move on to questions about the current depression care cascade in the [Insert Department] (See Page 2).

Please think about the process from screening, following up on a positive screen, referral, and linkage to care. For these questions, please also think about your role and what you have oversight over.

1. Based on a previous meeting we had with your clinic, we developed a visual of the care cascade. Let's walk through each step and I will ask you to describe your specific role.
    1. *Prompt for specific tasks after each step is reviewed.*

2. If you are not available to support any of the steps in the depression screening process, how might the medical team members within the [**Insert Department**] manage the workflow?

Challenges and Opportunities for Improvement

Take 5 minutes to think about the major challenges you face in your described roles as part of depression screening. Please have your top challenge ready to share.

3. Let's walk through your top challenge or pain point. For each, I'll ask you to propose one or more possible solutions that have worked or that you are newly suggesting.

- Let's start with the important challenge or pain point from your perspective?
    - Do you have suggestions or ideas about how to avoid or address this?
- Let's move on to another challenge? What is that?
    - Do you have suggestions or ideas about how to avoid or address this?

Continue this line of questioning until everyone has shared.

<u>Closing</u>*: "Thank you very much for sharing your valuable insight with me today. I will now stop recording."*
