# Supplementary material for: Team-Effectiveness Barriers and Solutions to Depression Screening within a Pediatric Hospital Network: A Qualitative Study
Source: Evid Based Pract Child Adolesc Ment Health. Author manuscript; Available in PMC 2026 Aug 11. (PMC13456526; doi:10.1080/23794925.2026.2711444)
Supplement: Supplementary file 2 [file NIHMS2200715-supplement-Supp_2.pdf]

Pre-Implementation
Focus Group: Clinic Name, Group #
Date: MM/DD/YYYY
Reviewer Initials:

	Questions							
	**Question 1:** Please share your name, role, and experience with depressions screening and linkage to care	**Question 2:** Please describe your current protocol for depression screening, linkage to care, and tracking outcomes.	**Question 3:** How would you describe a successful depression care cascade team?	**Question 3a.** What does communication look like in a successful depression care cascade team? *Probe for communication in Epic and via email or phone.*	**Question 3b.** What are the factors that make it more likely for team members to be on the same page versus not?	**Question 4:** Let's walk through your top challenge or pain point. For each, I'll ask you to propose one or more possible solutions that have worked or that you are newly suggesting.	**Question 4a:** Let's start with the important challenge or pain point from your perspective? *Do you have suggestions or ideas about how to avoid or address this?* **MAJOR CHALLENGES**	**Question 4b:** Let's move on to another challenge? What is that? *Do you have suggestions or ideas about how to avoid or address this?* **POSSIBLE SOLUTIONS**
**Responses**								
**Important Quotations**								
**Other Observations**								
